# Characteristics of replication-independent endogenous double-strand breaks in *Saccharomyces cerevisiae*

**DOI:** 10.1186/1471-2164-15-750

**Published:** 2014-09-01

**Authors:** Monnat Pongpanich, Maturada Patchsung, Jirapan Thongsroy, Apiwat Mutirangura

**Affiliations:** Department of Mathematics and Computer Science, Faculty of Science, Chulalongkorn University, Bangkok, Thailand; Program in Bioinformatics and Computational Biology, Graduate School, Chulalongkorn University, Bangkok, Thailand; Center for Excellence in Molecular Genetics of Cancer and Human Diseases, Chulalongkorn University, Bangkok, Thailand; Inter-Department Program of BioMedical Sciences, Faculty of Graduate School, Chulalongkorn University, Bangkok, Thailand; School of Medicine, Walailak University, Nakhon Si Thammarat, Thailand; Department of Anatomy, Faculty of Medicine, Chulalongkorn University, Bangkok, Thailand

**Keywords:** Replication-independent endogenous DNA double-strand breaks, RIND-EDSBs, Saccharomyces cerevisiae, Next-generation sequencing

## Abstract

**Background:**

Replication-independent endogenous double-strand breaks (RIND-EDSBs) occur in both humans and yeast in the absence of inductive agents and DNA replication. In human cells, RIND-EDSBs are hypermethylated, preferentially retained in the heterochromatin and unbound by γ-H2AX. In single gene deletion yeast strains, the RIND-EDSB levels are altered; the number of RIND-EDSBs is higher in strains with deletions of histone deacetylase, endonucleases, topoisomerase, or DNA repair regulators, but lower in strains with deletions of the high-mobility group box proteins or Sir2. In summary, RIND-EDSBs are different from pathologic DSBs in terms of their causes and consequences. In this study, we identified the nucleotide sequences surrounding RIND-EDSBs and investigated the features of these sequences as well as their break locations.

**Results:**

In recent work, we detected RIND-EDSBs using ligation mediated PCR. In this study, we sequenced RIND-EDSB PCR products of resting state *Saccharomyces cerevisiae* using next-generation sequencing to analyze RIND-EDSB sequences. We found that the break locations are scattered across a number of chromosomes. The number of breaks correlated with the size of the chromosomes. Most importantly, the break occurrences had sequence pattern specificity. Specifically, the majority of the breaks occurred immediately after the sequence “ACGT” (P = 2.2E-156). Because the “ACGT” sequence does not occur primarily in the yeast genome, this specificity of the “ACGT” sequence cannot be attributed to chance.

**Conclusions:**

RIND-EDSBs occur non-randomly; that is, they are produced and retained by specific mechanisms. Because these particular mechanisms regulate their generation and they possess potentially specific functions, RIND-EDSBs could be epigenetic marks.

**Electronic supplementary material:**

The online version of this article (doi:10.1186/1471-2164-15-750) contains supplementary material, which is available to authorized users.

## Background

In previous research, we detected endogenous DNA double strand breaks (EDSBs) that occurred without inductive agents or DNA replication and named them replication-independent EDSBs (RIND-EDSBs) [1–3]. The causes and consequences of RIND-EDSBs differ from those of replication-induced EDSBs [4] and irradiation-induced DSBs [5]. Whereas irradiation-induced DSBs halt the cell cycle [6], induce cell death [7] and serve as mediators of mutation [8], RIND-EDSBs do not. RIND-EDSBs are detectable in all human cell types [1] as well as in yeasts [3]. The down-regulation or deletion of high mobility group genes in both humans and yeasts lowers RIND-EDSB levels [3]. The high mobility group genes are multifunctional genes that regulate multiple DNA-dependent processes such as transcription, replication, recombination, and DNA repair [9, 10]. In conclusion, not only are RIND-EDSBs ubiquitously expressed in eukaryotic cells, but genes that maintain optimal levels of RIND-EDSBs also exist in cells. Therefore, RIND-EDSBs are evolutionally conserved in eukaryotic cells and may possess particular essential functions [1–3].

We previously reported that RIND-EDSBs may help to maintain genomic integrity. Firstly, RIND-EDSBs are linked to methylated CpG nucleotides [1], and genomic hypomethylation is associated with genomic instability [11–13]. Consequently, fragile genomes exhibit low levels of DNA methylation and also contain few RIND-EDSBs. Secondly, RIND-EDSBs are preferentially retained in the heterochromatin and are unbound by γ-H2AX [2]. Finally, when the number of RIND-EDSBs is reduced by trichostatin A, the number of γ-H2AX foci increases [2]. γ-H2AX foci are signals of pathologic DSBs [14, 15]. This suggests that RIND-EDSBs may prevent unwanted DNA breakage. Interestingly, the generation of RIND-EDSBs can be discovered in cells lacking topoisomerase [3]. Topoisomerase is involved in unwinding topological constraints by replication or transcription [16, 17]. Therefore, it is possible that RIND-EDSBs may help relieve DNA tension during transcription to prevent unwanted DNA breakage.

RIND-EDSBs do not result in mutations. We previously demonstrated that methylated RIND-EDSBs in human cells were repaired by a precise ATM-dependent type of non-homologous end joining (NHEJ) [2], whereas unwanted DSBs were generally repaired by a faster, more-error prone Ku-mediated type of NHEJ. A compact structure prevented H2AX phosphorylation, a conventional DSB repair response, which resulted in methylated RIND-EDSBs escaping error-prone NHEJ repair [2].

We now hypothesize that RIND-EDSBs are not pathologic DNA lesions and may have essential biological functions. Consequently, RIND-EDSBs should not occur randomly in genomes. In this study, we examined the characteristics of yeast RIND-EDSBs. First, the DNA sequences near the 3’ ends of RIND-EDSBs were amplified by ligation mediated PCR. Next, the resulting PCR products were sequenced using high throughput sequencing. Finally, these sequences were used to analyze the locations and sequences surrounding the RIND-EDSBs.

## Results

**Figure 1:**
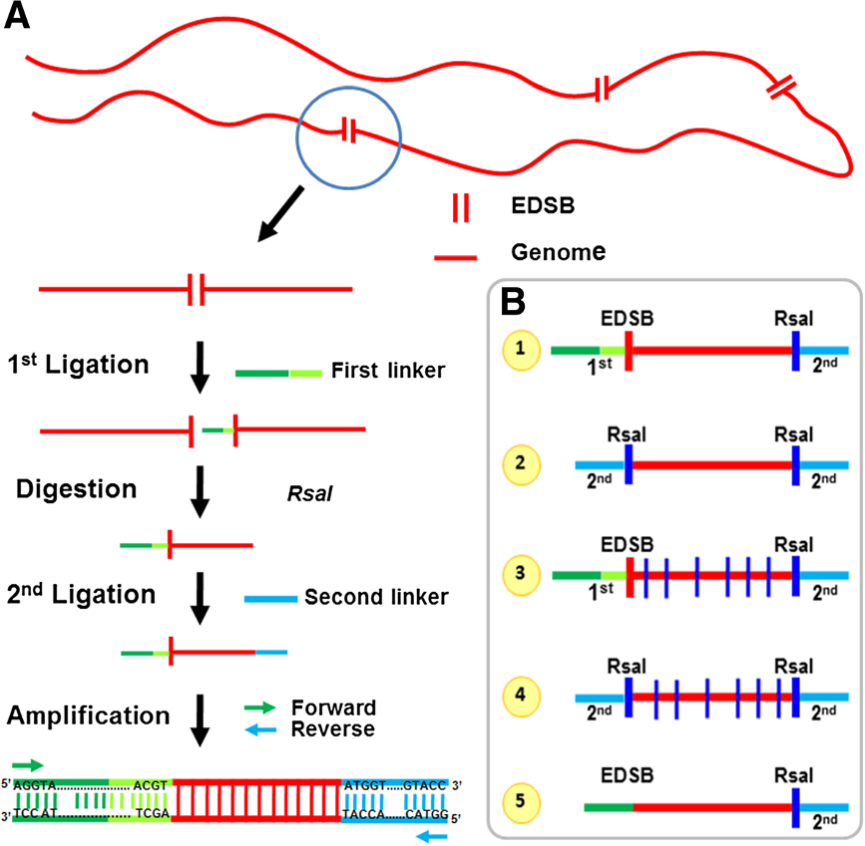
**Schematic representation of EDSB-detection using Ion Torrent sequencing. (A)** The red lines and parallel vertical bars represent genomic DNA and EDSB ends, respectively. The green line is the First linker sequence, where the darker shade represents the primer sequence. The blue line is the second linker sequence. The green and blue arrows represent the forward and reverse primers, respectively. In the first step, the First linker is ligated to one of the EDSB ends. Next, the DNA ligated to the first linker is digested with a restriction enzyme, RsaI, which does not cut between the linkers. After digestion, the second linker is ligated to the first linker-ligated DNA. Finally, the EDSBs were amplified with primers that bind to the first and second linkers. **(B)** The five PCR product patterns are shown. The red vertical bar represents the RIND-EDSBs whereas the blue vertical bar represents an RsaI break*.* (1) An intact sequence with both linkers. The RIND-EDSBs are between the First linker and the DNA sequence. (2) An intact sequence with Second linkers on both sides. There were no RIND-EDSBs here. (3) A sequence derived from multiple ligations with both of the linkers. (4) Multiple ligations with the Second linker only. (5) PCR products generated from nonspecific amplification due to the direct binding of the primers to the genome. These amplicons did not contain the 3’ sequence of the First linker and also lacked RIND-EDSBs.

The RIND-EDSB PCR products were prepared by ligation mediated PCR. First, a “First linker” was ligated to the RIND-EDSBs. Next, this ligated high molecular weight DNA was digested by RsaI, and the digested DNA was ligated to “Second linkers”. Then, a PCR was performed using primer sets with 5’ linker sequences (Figure 1A). Theoretically, there were five types of PCR products (Figure 1B). (1) Products that contained both of the linkers and the intact sequence, with the 5’ end of the intact sequence next to the First linker being RIND-EDSBs. (2) Products that contained the Second linkers on both sides and the intact sequence, but no RIND-EDSBs. (3) Products that contained both of the linkers and a sequence that was derived from multiple ligations. (4) Products that contained only the second linkers and multiple ligations. (5) PCR products that were generated from nonspecific amplification due to the primers binding directly to the genome. These amplicons lacked the 3’ sequence of the First linker and contain no RIND-EDSBs.

Because there was heterogeneity in the origins of the PCR products, we performed multiple steps to screen out PCR products that were not RIND-EDSBs or multiple ligation products (products 2–5). The first step was trimming the linkers, which allowed us to categorize the PCR products and retain types 1 and 3 (Additional file 1: Figure S1, step1). In 105 775 reads, there were 14 566 reads lacking the First linker (PCR product types 2 and 4), 69 229 reads containing the first linker sequence but missing ten or more of the last bases (PCR product type 5) and 21 980 reads containing both the first linker sequence and the last ten bases (PCR product types 1 and 3). Next, from PCR product types 1 and 3, we retained those reads having sequence lengths greater than 10 bases, and containing First linker sequences with 0 gaps and mismatches (Additional file 1: Figure S1, step 2). This step yielded 5 604 reads. Then, using only sequences (no linkers), the resultant reads were aligned to a BY4741 reference genome via BLAST (Additional file 1: Figure S1, step 3). In total, 1 283 (22.9%) of these reads were mapped to the reference genome by BLAST. Most of the sequences from the RIND-EDSB PCR products that were not mapped involved multiple ligations of RsaI digested fragments (PCR product type 3). In the control group, approximately 86% of the reads were mapped (notably, the control group did not use RsaI). RsaI cutting can yield small fragments. The smallest fragment size generated using RsaI is four bases. The distribution of possible fragment sizes after applying RsaI to BY4741 is shown in Additional file 1: Figure S2A and S2B. The most abundant fragment size was nine bases. However, if an RsaI cut site happens to be located near RIND-EDSBs, it can produce fragment sizes smaller than four bases. The distribution of potential fragment sizes after applying RsaI to BY4741, assuming the existence of RIND-EDSBs at “ACGT” sequences, is shown in Additional file 1: Figure S2C and S2D. The most abundant fragment size was 2 bases. These small fragments were ligated together randomly, thus producing reads that could not be mapped. However, multiple ligations did not interfere with the sequences with RIND-EDSBs at farther distances from RsaI cut sites. Using the BLAST results, we filtered out those reads that were not aligned from the first base (Additional file 1: Figure S1, step 4), resulting in 1085 (899 + 186) reads, where the two numbers in the parentheses are the number of uniquely mapped reads and multi-mapped reads, respectively. Finally, we examined the reads that were mapped to multiple locations (multi-mapped reads; Additional file 1: Figure S1, step 5). The final number of reads analyzed was 1080 (899 + 181) reads, where the two numbers in the parentheses are the number of uniquely mapped reads and multi-mapped reads, respectively. Over 97% of the multi-mapped reads were retained for analysis.

### Distribution and number of RIND-EDSBs

**Figure 2:**
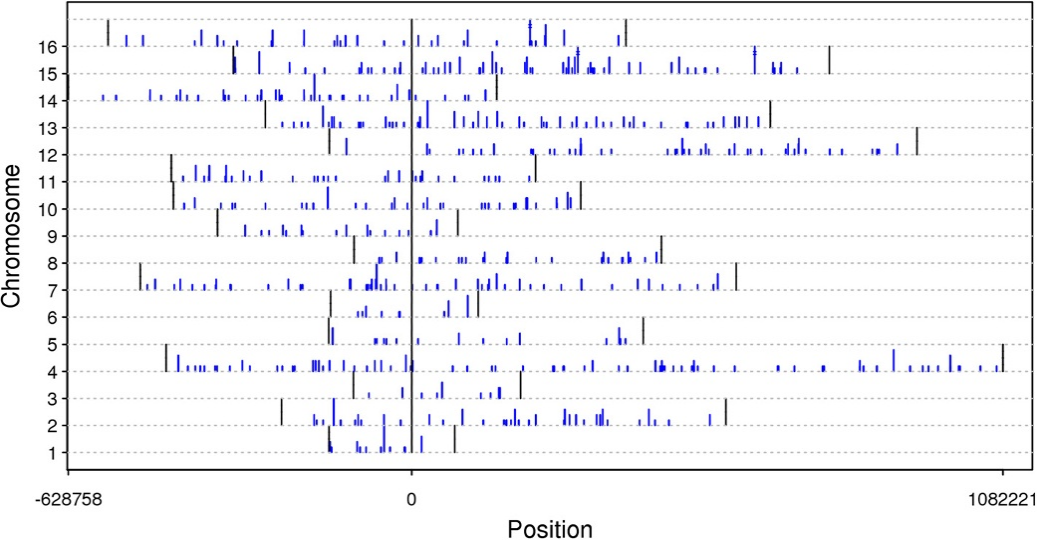
**Distribution of the RIND-EDSBs on each chromosome.** The x-axis corresponds to the chromosomal position given that position zero is the centromere (marked by a long vertical black line). The beginning and the end of each chromosome is marked by short vertical black lines. The y-axis represents the chromosome; chromosome 1 is at the bottom and chromosome 16 is at the top. The blue lines mark break positions. The height of each line represents the number of reads aligned to that position. Lines with two horizontal dashes across them indicate that there were more than five reads aligned to that position.

We investigated whether the break positions had any relationship with chromosomal positions, e.g., with centromeric or telomeric regions. The alignment positions from BLAST corresponded to break positions; thus, we were able to observe the EDSB distribution across all 16 chromosomes. In the wild type yeast cells, 899 uniquely mapped reads corresponded to 729 positions in the genome. That is, for some positions, multiple reads were mapped because they originated from a population of cells rather than from a single cell. Figure 2 shows the 729 EDSB positions. The height of each vertical bar corresponds to the number of reads aligned to that specific position. As shown in the figure, the break positions did not clump near the centromeres or the telomeres but were scattered across the chromosomes.

**Figure 3:**
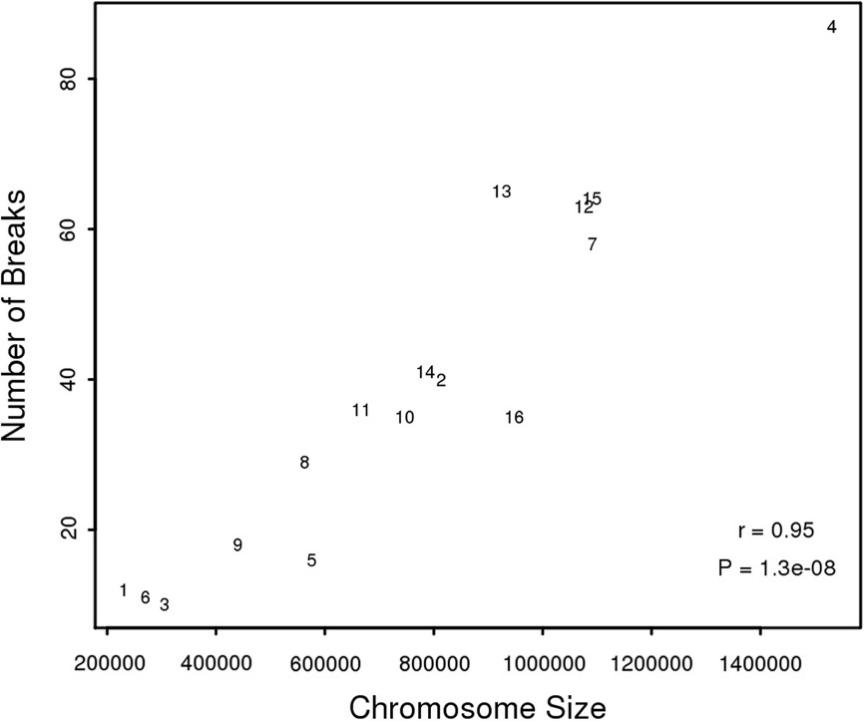
**Number of breaks and chromosomal size.** The x-axis represents chromosomal size and the y-axis represents the number of breaks on the chromosome. The numbers in the graph correspond to chromosome numbers.

To see if the number of breaks correlated with chromosomal size, we plotted the number of breaks against the length of the chromosomes (Figure 3). The number of breaks increased nearly linearly with chromosomal size. The Pearson correlation coefficient for this observation was 0.95, and the p-value was 1.3E-08.

### Sequence patterns near break points

**Figure 4:**
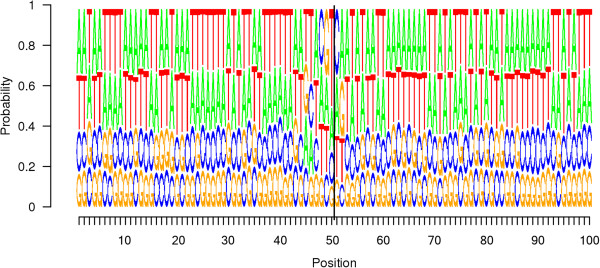
**Sequence logo.** The sequence logo where all of the columns have the same height. The break is located between positions 50 and 51 and is marked with a vertical line.

To explore whether specific sequence patterns existed near the breaks, we plotted sequence logos (Figure 4 and Additional file 1: Figure S3) of 1,080 reads (see the Sequence logo section in the Methods section). In Figure 4, we observed a difference in the base frequency patterns between columns 45 to 52, which spanned across the breakpoints. In other columns, we can see that the frequency of C is similar to that of G, the frequency of A is similar to that of T and the frequencies of C and G are generally lower than the frequencies of A and T. This is consistent with the proportions of A, C, G and T in the yeast genome, which are 30.9%, 19.2%, 19.1% and 30.8%, respectively. Additional file 1: Figure S3 shows sequence logos where the column height is proportional to the information content. Although the overall information content in each column is low, we can see that position 50, located right before a breakpoint, has the highest information content, which indicates a lower tolerance for substitutions at this position compared with others.

We next investigated whether break occurrences have specificities toward certain sequences. Because base frequency patterns differ between columns 45–52, we counted, in a wild type yeast sample, the number of stitched reads with base combinations and column positions as follows: “xxx^▼^x”, “xxxx^▼^”, “xxxx^▼^x”, “xxxx^▼^xx”, “xxxxxx^▼^”, “xxxxxx^▼^xx”, where x denotes a single base and “^▼^” denotes a break (see the *Read counting* section in the Methods section). We show the counts for each sequence pattern in Additional file 2. Regardless of the sequence type, we observed a non-uniform distribution of counts where one base combination had the highest counts of all the base combinations. This highest count was for the “xxxx^▼^” combination compared with the “xxx^▼^x”, “xxxx^▼^x”, “xxxx^▼^xx”, “xxxxxx^▼^”, and “xxxxxx^▼^xx” combinations. This implies that the breaks were more likely to occur at certain sequences. To determine whether this sequence specificity was statistically significant, we performed a Fisher’s exact test (see the Statistical analysis section in the Methods section). Of the 1 080 stitched reads, we observed that 124 of them contained “ACGT^▼^” at the breakpoints. In the BY4741 genome, we observed that “ACGT” occurred 29 319 times, whereas other 4-mer sequences occurred 11 757 514 times. The associated p-value was 2.2E-156, which was highly significant (Table 1, first row and Additional file 3).

**Table 1:** **Fisher’s exact test p-values**

4-mer	A*	B*	C*	D*	Adjusted p-value	Odds ratio	95% CI
ACGT	124	956	29319	11757514	2.20E-156	51.96	42.76, 62.89
TAGT	19	1061	44649	11742184	8.45E-06	4.71	2.82, 7.39
CAGT	17	1063	41209	11745624	4.15E-05	4.56	2.64, 7.34
TGGT	18	1062	53437	11733396	2.43E-04	3.72	2.20, 5.91
TACC	13	1067	40441	11746392	5.45E-03	3.54	1.88, 6.08
ATAT	24	1056	91778	11695055	3.85E-04	2.90	1.85, 4.33
XXXX^1^	0	1080	1112372	10674461	4.79E-47	0.00	0.00, 0.03

*See the description in the Methods section.

^1^combined 4-mer sequences that were never observed in the reads.

We further validated the non-random occurrence of “ACGT^▼^” sequences using the simulations described in the Methods section. In contrast with the observed data, the 100 sequence logos from the simulated data had no specific patterns in any of the columns. In every column of the logos, C and G, whose frequencies are approximately equivalent, have lower frequencies than A and T, which have similar frequencies. This frequency pattern is the same for all of the columns in the observed data, with the exception of the “ACGT^▼^” columns. This further supports our conclusion of a nonrandom association of “ACGT^▼^” sequences with breaks.

**Figure 5:**
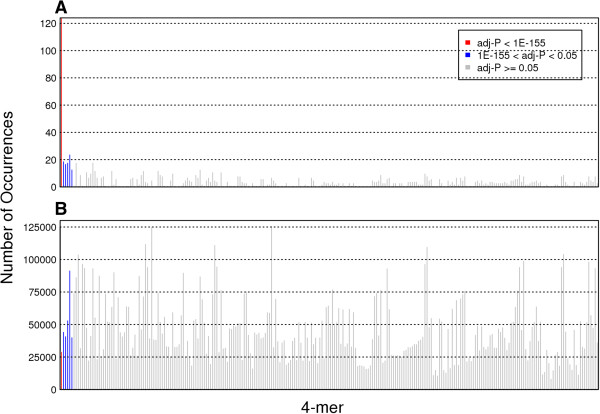
**Number of 4-mer sequences. (A)** The number of stitched reads with each “xxxx^▼^” 4-mer sequence. **(B)** The number of locations in the genome with each “xxxx^▼^” 4-mer sequence. The x-axis represents all 256 possible 4-mer sequences, and the 4-mers are sorted by adjusted p-values (Additional file 3). The y-axis represents the occurrence of each 4-mer sequence.

To examine whether the breaks were associated with other 4-mer sequences, we calculated a Fisher’s exact test and an odds ratio for the other possible 4-mer sequences (Additional file 3). The significant 4-mer sequences are shown in Table 1. In addition to “ACGT^▼^”, the p-values of “TAGT^▼^”, “CAGT^▼^”, “TGGT^▼^”, “TACC^▼^”, and “ATAT^▼^” were significant with non-zero odds ratios. We plotted the number of stitched reads with each possible 4-mer sequence preceding the breaks, “xxxx^▼^” (Figure 5A), as well as the number of each 4-mer occurrence in the yeast genome (Figure 5B). Comparing the two figures clearly illustrates that the significant 4-mer sequences common in the reads were not predominant in the genome. These results lead us to conclude that RIND-EDSBs are more likely to occur at specific sequence patterns.

To determine if multi-mapped reads influenced the break preference at “ACGT”, we retained uniquely mapped reads only (899 reads) and counted all of the possible 4-mer sequence of “xxxx^▼^” in the stitched reads as we had done previously. The breaks generally occurred before “ACGT^▼^” as they had before (Table 2; Additional file 4).

**Table 2:** **The top ten highest occurrences of the “xxxx▼” 4-mer in the stitched reads (uniquely mapped reads only)**

4-mer	Count	Proportion
ACGT	113	0.13
ATAT	23	0.03
TTCT	18	0.02
CAGT	17	0.02
TTAT	17	0.02
TAGT	15	0.02
TACC	12	0.01
TGGT	12	0.01
AAGT	12	0.01
GGTT	10	0.01

We also tested whether our PCRs and/or methodology generated any artifacts resulting in biased results by using AluI digested DNA as a control (see the Methods section). Almost all of the cut sites in the PCRs at both 20 and 60 cycles were expected, that is, we observed “AG^▼^CT” in our stitched reads (Tables 3 and 4; Additional file 5). Therefore, it is unlikely that our RIND-EDSB results were biased.

**Table 3:** **The top ten highest occurrences of the “xx** ^**▼**^ **xx” 4-mer in the stitched reads of control group I**

4-mer	Count	Proportion
AGCT	251611	9.989E-01
GTAC	171	6.788E-04
GTCT	24	9.528E-05
CGCT	11	4.367E-05
CTCT	10	3.970E-05
GTCA	5	1.985E-05
AACT	5	1.985E-05
GTAA	4	1.588E-05
CAGC	4	1.588E-05
GAGC	4	1.588E-05

**Table 4:** **The top ten highest occurrences of the “xx** ^**▼**^ **xx” 4-mer in the stitched reads of control group II**

4-mer	Count	Proportion
AGCT	100732	9.984E-01
GTAC	64	6.343E-04
GTCT	20	1.982E-04
CGCT	16	1.586E-04
CTCT	5	4.955E-05
ATCT	5	4.955E-05
TTCT	5	4.955E-05
TCCT	4	3.964E-05
GTCA	3	2.973E-05
ACCT	3	2.973E-05

**Figure 6:**
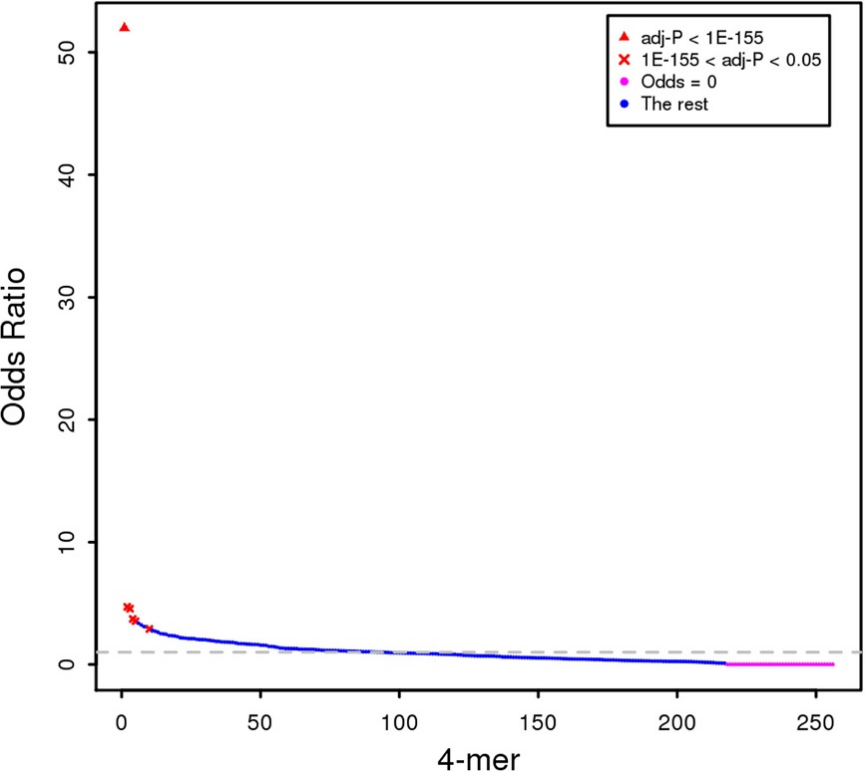
**Sorted odds ratios of each 4-mer.** The x-axis represents all 256 possible 4-mer sequences sorted by odds ratios (Additional file 3: Table S2). The y-axis represents the odds ratios. The red triangles denote the significant 4-mer sequences with odds ratios > 10. The red ‘x’ denote the significant 4-mer sequences with odds ratios < 10. The pink dots denote the 4-mer sequences with odds ratios equal to zero. The horizontal dotted grey line indicates an odds ratio equal to 1.

**Figure 7:**
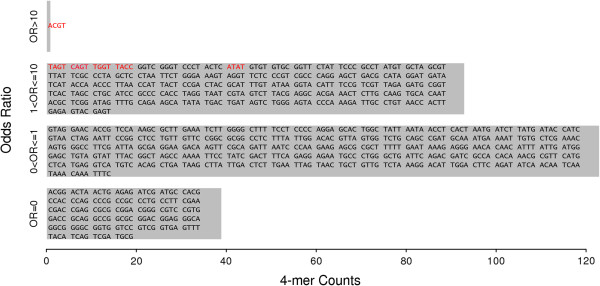
**The number of 4-mer sequences in each range of odds ratios.** The y-axis represents four ranges of odds ratios starting at the bottom of the graph and going upwards: OR = 0, 0 < OR ≤ 1, 1 < OR ≤ 10 and OR > 10. The x-axis represents the number of 4-mers per range. The sequences of each 4-mer in the range are shown; those shown in red are significant.

In conclusion, the most frequent sequence before breaks was observed to be “ACGT”, which had an exceptionally high odds ratio (the red triangles in Figure 6). Moreover, there were additional 4-mer sequences that occurred frequently with odds ranging from 2.9-5 (the red ‘x’ in Figure 6). This group of 4-mers often contained “GT” before breaks. Conversely, some 4-mer sequences had odds of zero (the pink dots in Figure 6). These 4-mer sequences were never observed in the reads. When these zero odds 4-mers are put together, the p-value is extremely significant (4.8E-47; the last row in Table 1). This finding demonstrates that break occurrences exhibit sequence specificity, i.e., there are no breaks at these 4-mer sequences. The number of 4-mer sequences with no breaks was not trivial (Figure 7).

## Discussion

We postulated that RIND-EDSBs have an important function in cells, and therefore their occurrence is nonrandom. In this study, we examined the characteristics of RIND-EDSBs to determine if RIND-EDSBs occur systematically. We found that breaks were scattered along chromosomes and that the number of breaks and chromosomal size were positively and almost linearly correlated. Most importantly, the breaks occurred most frequently after the sequence “ACGT”.

RIND-EDSBs are evolutionarily conserved, i.e., they are present in both the human and yeast genomes. In addition, there are lower numbers of RIND-EDSBs in yeast cells depleted of Nhp6a. These same results occurred in human cells lacking Hmgb1, the human homolog of Nhp6a [3].

We speculate that there is a specific mechanism for the occurrence of RIND-EDSBs; however, this mechanism remains unknown. It might resemble the mechanism used by restriction enzymes to recognize and cut a specific nucleotide sequence or it might depend on the physical properties of dinucleotide sequences. The specificity of “ACGT” was not a coincidence because “ACGT” was ranked number 189 out of 256 with respect to “xxxx” abundance in the genome. In addition, its sequence logo implied that breaks preferred regions where a CG dinucleotide was in the neighborhood of an A or T. In other words, the physical properties of the CG dinucleotide in the ACGT sequence are distinctive from the physical properties of the surrounding nucleotides. It is unlikely that the mechanism is dependent on chromosomal location because we observed that break locations were scattered across chromosomes, suggesting that there are distinct RIND-EDSB locations in each cell. Because RIND-EDSBs have been conserved from yeasts to humans, the mechanism by which RIND-EDSBs are produced should have been conserved as well.

RIND-EDSBs are potential epigenetic marks. Epigenetic marks require two properties. Firstly, they must be associated with a non-random underlying mechanism. For example, DNA methylation is specific to the cytosine in CpG dinucleotides due to the DNA methyltransferase function [18, 19]. Secondly, an epigenetic mark must specifically function to control gene expression or affect genomic integrity. For example, methylation in promoter suppressed gene expression [20, 21], intragenic LINE-1 hypomethylation reduced gene expression [22], and hypomethylation can all lead to genomic instability [11]. Due to the conservation of RIND-EDSBs in every cell from yeasts to humans, we deduced that a specific mechanism must exist to produce RIND-EDSBs. Moreover, in human cells, γ-H2AX, which is a marker of abnormal DNA breakage, was up-regulated when concentrations of RIND-EDSBs were reduced [2]. When HMGB1 was depleted, the levels of RIND-EDSBs decreased, suggesting a role for HMGB1 in maintaining RIND-EDSBs [3]. A yeast strain lacking the genes involved in generating DSBs, e.g., topoisomerases or endonucleases, had an increased level of RIND-EDSBs [3]. This implies that there is a compensatory mechanism for the loss of the functions of certain topoisomerases or endonucleases, and indicates a potential role for RIND-EDSBs. Here in this study we proved that RIND-EDSB occurring non-randomly implying the association with a non-random underlying mechanism. Therefore, we provided additional information to support that RIND-EDSBs are epigenetic marks.

## Conclusions

In summary, we found that RIND-EDSBs were location-independent and tended to occur immediately after the sequence “ACGT”. This finding suggests that RIND-EDSB production is regulated by a non-random mechanism. The fact that RIND-EDSBs have a specific mechanism and are retained for a specific function signifies that RIND-EDSBs are epigenetic marks.

## Methods

### Yeast strains, media and growth conditions

The wild type yeast *Saccharomyces cerevisiae* strain BY4741 (*MATa his3∆1 leu2∆0 met15∆0 ura3∆0*), was used. The yeast strain was grown at 30°C in 10 ml of liquid YPD media (Sigma, USA) for 1 day to the stationary phase (~8 × 10^6^ cells/mL) and diluted to 1 × 10^4^ cell/mL in YP medium containing 2% raffinose (Sigma, USA) for 48 hours (for synchronization). Cells in the G0 phase were determined by their morphology and counted under a microscope with a hemacytometer. The percentage of G0 phase cells was calculated using the following equation: (number of G0 phase cells/total number of cells) × 100. The wild type yeast strain (BY4741) consisted of 100% unbudded stationary-phase cells. Examples of budded and unbudded yeast cells are shown in Additional file 1: Figure S4. Whole cells were pelleted by centrifugation at 5,000 rpm for 5 minutes.

### High-molecular weight (HMW) DNA preparation

The yeast cell pellets were treated with 1 mg/ml lyticase (70 U/mg) (Sigma, USA) for 2 hours and embedded in 1% low melting point agarose (MO BIO, USA) at a concentration of 2x10^8^ cells per plug mold. The plugs with cells were then digested in 400 μl of digestion buffer (1 mg/ml proteinase K, 50 mM Tris, pH 8.0, 20 mM EDTA, 1% sodium lauryl sarcosine) at 37°C overnight. The plugs were washed 6 times in TE buffer for 40 minutes, and the cohesive end EDSBs were polished with T4 DNA polymerase (New England Biolabs, USA). T4 DNA polymerase was inactivated by incubation in 20 mM EDTA for 5 minutes followed by 6 washes in TE buffer for 40 minutes. The First linkers were prepared from the two oligonucleotides 5′-AGGTAACGAGTCAGACCACCGATCGCTCGGAAGC TTACCTCGT GGACGT-3′ and 5′-ACGTCCACGAG-3′ (Sigma, Singapore) [1] for ligation to the HMW DNA using T4 DNA ligase (New England Biolabs, USA) at 25°C for 2 nights. The DNA was extracted from the agarose plugs using a QIAquick gel extraction kit (Qiagen, Switzerland) [1]. The DNA was digested in a 100 μL reaction volume with 2 U of RsaI in 1X NEB buffer (New England Biolabs, USA) [4] at 37°C overnight. The digested DNA was purified with phenol:chloroform and ethanol precipitations and ligated again with the second linkers (50 μM), which were prepared from the two oligonucleotides 5′ATGGTACCACCCGTAGGCCCTAC CGGT ACC -3′ and 5′-GGTACCGGTAGGGCCTACGGGTGGTACCAT -3′ (Sigma, Singapore). The ligated DNA was purified with a QIAquick PCR Purification Kit (Qiagen, Switzerland).

We have shown previously that the detection of the RIND-EDSBs was not due to our DNA preparation protocol [3].

### Library preparation and sequencing

Our principal method of RIND-EDSB detection is summarized in Figure 1A. After ligation with the two linkers, the HMW DNA was subjected to 60 cycles of PCR with two primers, First-L-F (5′-AGGTAACGAGTCA GACCACCGA-3′) and Second-L-R (5′- GGTACCGGTAG GGCCTACGGGT-3′ (Sigma, Singapore) using an annealing temperature of 65°C. The PCR product was purified with a QIAquick PCR Purification Kit (Qiagen, Switzerland) and sequenced on an Ion Torrent sequencer (Ion Torrent™ Personal Genome Machine® (PGM), Life Technologies, USA).

### Standard control sequencing method

We used the wild type yeast BY4741 strain as a standard for the real-time PCR. To validate our method for identifying clusters of breaks, we also sequenced this standard on an Ion Torrent sequencer. The yeast strain was grown at 30°C in 10 ml of liquid YPD media (Sigma, USA) for 1 day to the stationary phase (~8 × 10^6^ cells/mL) and diluted to 1 × 10^4^ cell/mL in YP medium containing 2% raffinose (Sigma, USA) for 48 hours (for synchronization). The cell cycle phases were confirmed by phase-contrast microscopy. The whole cells were pelleted by centrifugation at 5,000 rpm for 5 minutes. The pelleted cells were digested in 400 μl of digestion buffer (1 mg/ml proteinase K, 50 mM Tris, pH 8.0, 20 mM EDTA, 1% sodium lauryl sarcosine) at 37°C overnight. The DNA was extracted using phenol chloroform and ethanol precipitations. The DNA (5 μg) was digested with 2 U of AluI (New England Biolabs, USA) at 37°C overnight. The digested DNA was purified using phenol: chloroform and ethanol precipitations and ligated with the first linkers prepared from the two oligonucleotides 5′-AGGTAACGAGTCAGACCACCGATCGCTC GGAAGCTTACCTCGTGGACGT-3′ and 5′-ACGTCCACGAG-3′. The ligated DNA was purified with a QIAquick PCR Purification Kit (Qiagen, Switzerland). The ligated DNA was ligated with the second linkers (50 μM), which were prepared from the two oligonucleotides 5′ATGGTACCACCCGTAGGCCCTAC CGGT ACC -3′ and 5′-GGTACCGGTAGGGCCTACGGGTGGTACCAT -3′ (Sigma, Singapore). The ligated DNA was purified with a QIAquick PCR Purification Kit. After ligation with the two linkers, the HMW DNA was subjected to 20 (control I) and 60 cycles (control II) of PCR with two primers, First-L-F (5′-AGGTAACGAGTCA GACCACCGA-3′) and Second-L-R (5′- GGTACCGGTAG GGCCTACGGGT-3′ (Sigma, Singapore) using an annealing temperature of 65°C. The PCR product was purified with a QIAquick PCR Purification Kit (Qiagen, Switzerland) and sequenced on an Ion Torrent sequencer (Ion Torrent™ Personal Genome Machine® (PGM), Life Technologies, USA).

### Data processing and mapping

Each sequenced read contained different linkers at both ends of the read; therefore, we had to trim the linkers from both ends of the reads (Additional file 1: Figure S1, step1). We refer to the linkers that were ligated to the beginning and end of the reads as the First and Second linkers, respectively. We trimmed the linkers by aligning the First and Second linker sequences against the read sequences using the “pairwiseAlignment” function (we rewarded 1 for a match, and penalized 2, 5 and 2 for a mismatch, a gap opening and a gap extension, respectively) of the Biostrings package [23] in R. It was unclear whether the reads came from the plus or minus strands; therefore, we aligned the First and Second linkers to the reads from the fasta files and the reverse complement sequences. If a read was from the plus strand, the alignment against the original sequence was closer, whereas a minus strand read aligned more closely with the reverse complement. An alignment was considered successful if it met three criteria. (1) The aligned length was greater than 10 bases but no longer than the linker length. (2) The last aligned base position of the First linker was within the First linker length. A similar logic was applied to the Second linker. Because linkers should be aligned at the ends of reads, if the aligned position was beyond the linker length counting inwards from the end, we did not have confidence in the alignment. (3) The last ten bases of the First linker sequence must match. Because break positions were located between the last base of the First linker sequence and its next base, we could be more certain that break positions were accurate if the bases in the tail region of the linkers were aligned. Ten was arbitrarily chosen as the number of matched bases. We put no similar restrictions on the Second linker.

The trimmed reads with lengths longer than 10 bases and First linker alignments containing 0 gaps and mismatches were retained (Additional file 1: Figure S1, step 2) and aligned (Additional file 1: Figure S1, step 3) using Nucleotide-Nucleotide BLAST 2.2.27+ [24] with the default parameters against the *S. cerevisiae* strain BY4741 genome, which was downloaded from the Saccharomyces Genome Database (SGD).

From the BLAST results, we retained only those alignments that were mapped from the first base of the sequence (Additional file 1: Figure S1, step 4). In addition, some reads were aligned to many locations (multi-mapped reads). Because we were able to retrieve bases prior to each mapped position, we retained a multi-mapped read if more than 90% of the mapped positions had the same prior four bases (Additional file 1: Figure S1, step 5). After the completion of all of these steps, we produced a final set of reads in each sample for further analysis.

### Data analysis

#### Sequence logo

We extracted the first 50 bases of each read in the final set of reads and added the preceding 50 bases of the sequences, which were obtained from the reference genome. We refer to these reads as stitched reads. The preceding 50 bases were taken from the 5′ direction of the same strand (plus or minus) to which the read was mapped. Therefore, the break positions were located between the 50th and 51st bases. Because each read had a variable length, some reads were shorter than 50 bases, and thus the stitched read sequence lengths were not always 100. Consequently, the number of reads used to plot each column of the sequence logo is different (shown in Additional file 6). The height of each sequence logo column is proportional to its information content; however, we also plotted a variation of the sequence logo where all of the columns have the same height. The package “seqLogo” was used for plotting [25].

#### Sequence logo simulations

We sampled chromosomal position numbers with replacement for simulation purposes for each chromosome. The number of positions sampled was the observed number of breaks on the chromosome. Strands were assigned to each sampled number by sampling from 0 and 1 with a probability equal to the proportion of the reads mapped to the plus strand in each chromosome. The sampled numbers were regarded to be read alignment positions.

To perform the sequence logo simulations, we extracted the sequence starting 50 bases to the left of the sampled position and extending 49 bases to the right of the sampled position from the reference genome and plotted the sequence logos based on these extracted sequences.

#### Read counting

Let x denote a single base and let “^▼^” denote a break. Note that “^▼^” is between positions 50 and 51 in a stitched read. We introduce the following notations: “xxx^▼^x”, “xxxx^▼^”, “xx^▼^xx”, “xxxx^▼^x”, “xxxx^▼^xx”, “xxxxxx^▼^”, and “xxxxxx^▼^xx”. “xxx^▼^x” represents a specific combination of three bases before a break and one specific base after a break. There were 256 possible combinations of bases (i.e., AAA^▼^A, AAA^▼^C,…, TTT^▼^T). “xxxx^▼^” represents a specific combination of four bases before a break. There were 256 possible combinations of bases (i.e., AAAA^▼^, AAAC^▼^,…, TTTT^▼^). A similar logic can be used to interpret the remaining notations. We counted the occurrences of “xxx^▼^x”, “xxxx^▼^”, “xxxx^▼^x”, “xxxx^▼^xx”, “xxxxxx^▼^”, and “xxxxxx^▼^xx” in the stitched reads of the wild type sample and counted the number of occurrences of “xx^▼^xx” in the stitched reads of the control I and II samples. Additional file 7 (the “Count occurrences in stitched reads” section) shows a toy example.

In addition, we counted the “xxxx” occurrences in the reference genome, where “xxxx” indicates a specific combination of four bases. Additional file 7 (the “Count occurrences in genome” section) shows a toy example.

#### Statistical analysis

We constructed 256 2x2 contingency tables for each sample. For each table, the rows consist of stitched reads vs. the genome, and the columns contain one specific combination of “xxxx” occurrences vs. the rest of the combinations of “xxxx”. The first column of the first row, denoted by A, corresponds to the number of stitched reads that had one combination of “xxxx^▼^”. The second column of the first row, denoted by B, corresponds to the number of stitched reads that had the remainder of the combinations of “xxxx^▼^”. In the second row, the first column, denoted by C, contains the number of positions in the genome that had one combination of “xxxx”. The second column of the second row, denoted by D, contains the number of positions in the genome that had the remainder of the combinations of “xxxx”. We then computed a Fisher’s exact test p-value from these contingency tables. We used FDR to correct for multiple testing (using the R stats package, which is part of R; [26]). Additional file 7 (“Contingency table” section) shows a toy example.

### Availability of supporting data

The raw sequencing data have been deposited in the DDBJ Sequence Read Archive (DRA) under the accession number DRA002436.

## Electronic supplementary material

Additional file 1: Figure S1: data processing workflow. Number 1-5 in yellow circles represent five types of PCR product (see Figure 1B). There are five steps in the workflow: (1) Trim linkers which resulted in 3 categories of PCR products. (2) Retain reads of length greater than 10 bases and contain no mismatch and no gaps in the First linker. (3) BLAST the retained reads. (4) Retain reads from BLAST results that are aligned from the first base. (5) Retain multi-mapped reads if more than 90% of its mapped positions had the same prior four bases. **Figure S2.** histogram of possible fragment size. (A) all possible fragment size from using RsaI on BY4741 genome (B) zoom in version of A (C) all possible fragment size from using RsaI on BY4741 genome that contain RIND-EDSBs by assuming RIND-EDSBs occurred at ACGT (D) zoom in version of C. **Figure S3.** sequence logo. Sequence logo where column height is proportional to its information content. The break is between position 50 and 51 marked by vertical line. **Figure S4.** the morphology of wide type yeast strain. (A) The morphology of budding yeast cells in YPD media. (B) The morphology of unbudded stationary-phase cells in YP medium containing 2% raffinose. (PDF 275 KB)

Additional file 2: Table S1: Occurrences of each sequence pattern in the wild type yeast sample (including multi-mapped reads). (XLSX 1 MB)

Additional file 3: Table S2: Fisher’s exact tests and odds ratios. (XLSX 61 KB)

Additional file 4: Table S3: Occurrences of each sequence pattern in the wild type yeast sample (uniquely mapped reads only). (XLSX 1 MB)

Additional file 5: Table S4: Occurrences of each “xx^▼^xx” 4-mer in the stitched reads of the control groups I and II. (XLSX 18 KB)

Additional file 6: Tables S5: The number of reads contributing to the sequence logo plotting in each column. (XLSX 12 KB)

Additional file 7: **Toy examples for counting the occurrences in the stitched read, genome, and contingency tables.** (PDF 119 KB)

## Abbreviations

EDSBs: Endogenous double-strand breaks
RIND-EDSBs: Replication-independent EDSBs
NHEJ: Non-homologous end joining.
