# Supplementary material for: Characteristics of replication-independent endogenous double-strand breaks in Saccharomyces cerevisiae
Source: BMC Genomics. 2014 Sep 1;15(1):750. doi: 10.1186/1471-2164-15-750 (PMC4158086; doi:10.1186/1471-2164-15-750)
Supplement: Supplementary file 1 — Additional file 1: Figure S1: data processing workflow. Number 1-5 in yellow circles represent five types of PCR product (see Figure 1B). There are five steps in the workflow: (1) Trim linkers which resulted in 3 categories of PCR products. (2) Retain reads of length greater than 10 bases and contain no mismatch and no gaps in the First linker. (3) BLAST the retained reads. (4) Retain reads from BLAST results that are aligned from the first base. (5) Retain multi-mapped reads if more than 90% of its mapped positions had the same prior four bases. Figure S2. histogram of possible fragment size. (A) all possible fragment size from using RsaI on BY4741 genome (B) zoom in version of A (C) all possible fragment size from using RsaI on BY4741 genome that contain RIND-EDSBs by assuming RIND-EDSBs occurred at ACGT (D) zoom in version of C. Figure S3. sequence logo. Sequence logo where column height is proportional to its information content. The break is between position 50 and 51 marked by vertical line. Figure S4. the morphology of wide type yeast strain. (A) The morphology of budding yeast cells in YPD media. (B) The morphology of unbudded stationary-phase cells in YP medium containing 2% raffinose. (PDF 275 KB) [file 12864_2014_6418_MOESM1_ESM.pdf]

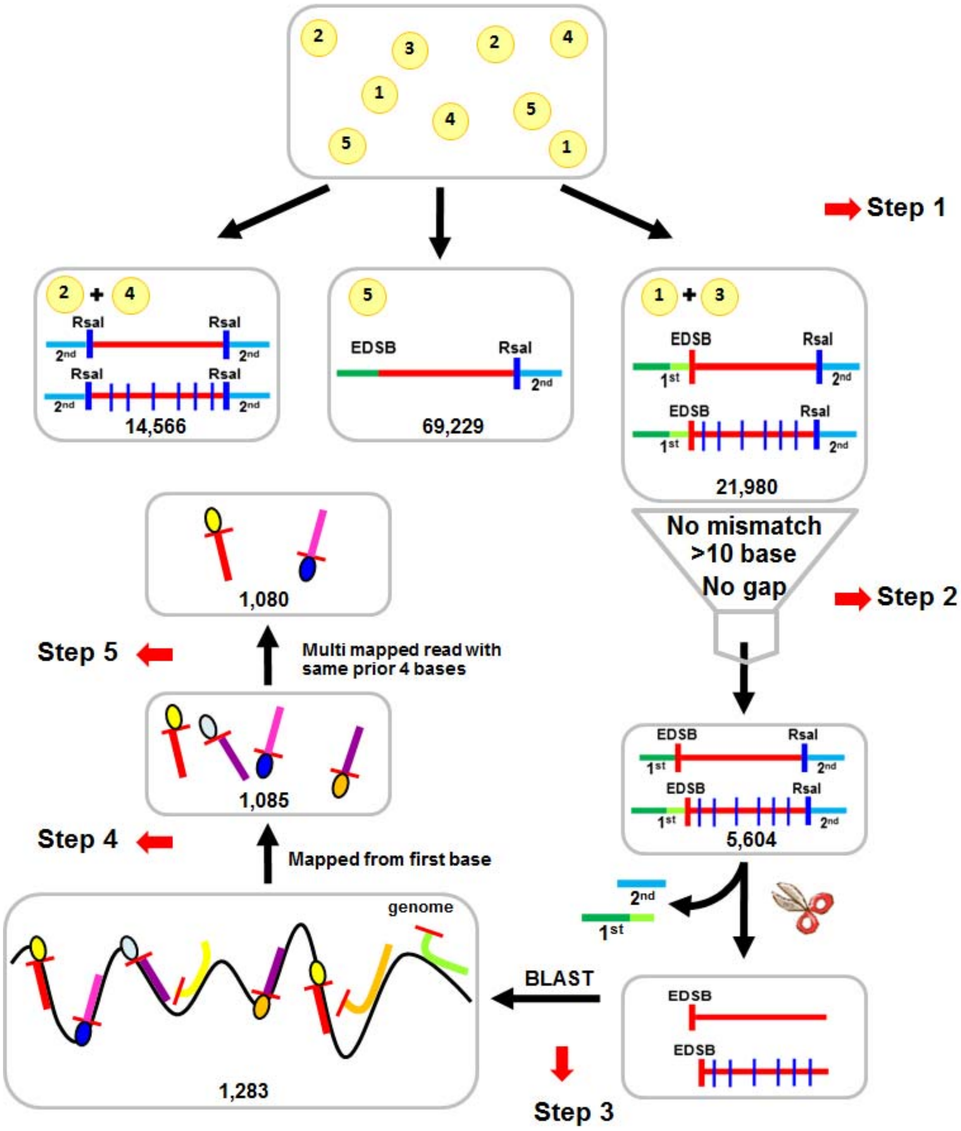

Figure S1. **Data processing workflow**. Number 1-5 in yellow circles represent five types of PCR product (see Figure 1B). There are five steps in the workflow: (1) Trim linkers which resulted in 3 categories of PCR products. (2) Retain reads of length greater than 10 bases and contain no mismatch and no gaps in the First linker. (3) BLAST the retained reads. (4) Retain reads from BLAST results that are aligned from the first base. (5) Retain multi-mapped reads if more than 90% of its mapped positions had the same prior four bases.

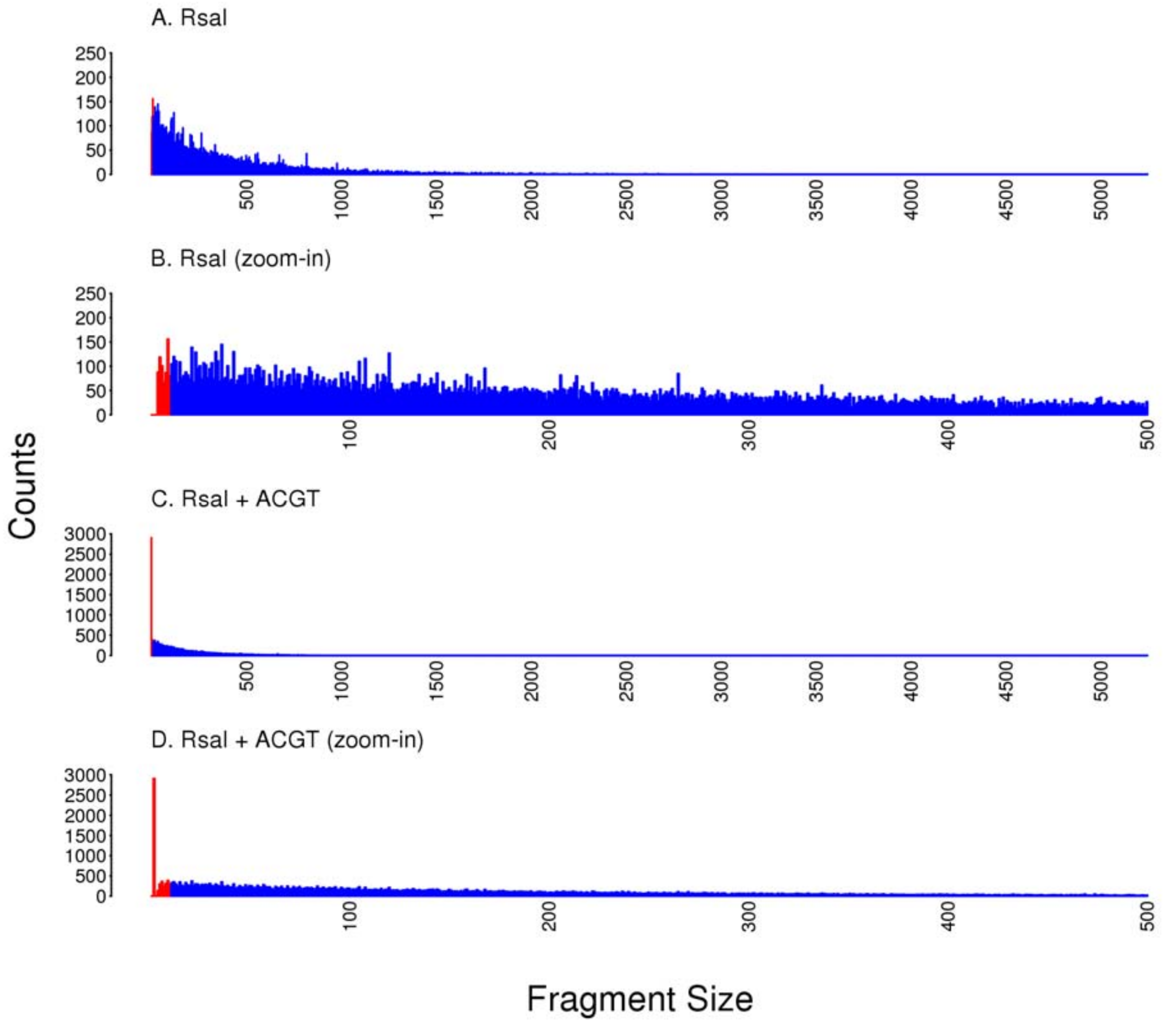

Figure S2. **Histogram of possible fragment size.** (A) all possible fragment size from using RsaI on BY4741 genome (B) zoom in version of A (C) all possible fragment size from using RsaI on BY4741 genome that contain RIND-EDSBs by assuming RIND-EDSBs occurred at ACGT (D) zoom in version of D

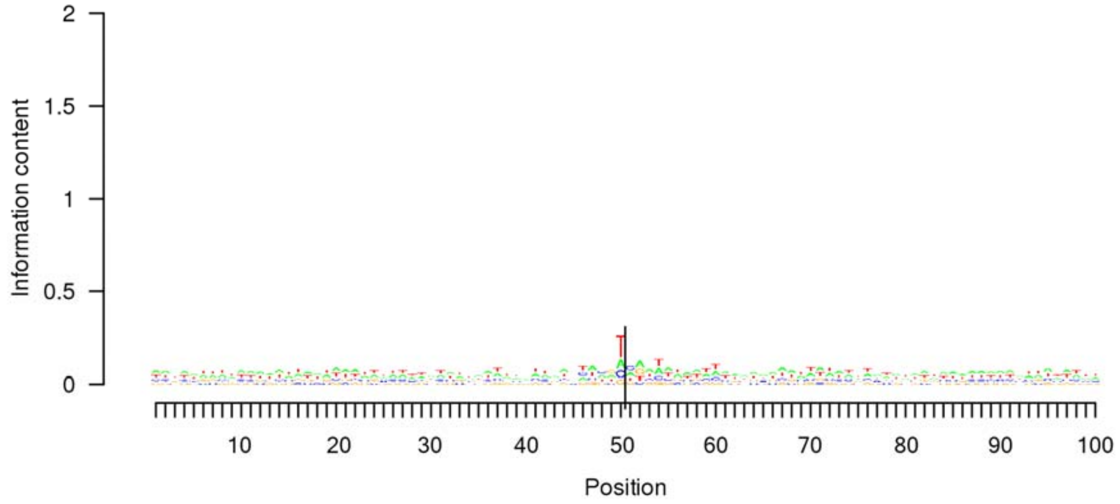

Figure S3. **Sequence logo.** Sequence logo where column height is proportional to its information content. The break is between position 50 and 51 marked by vertical line.

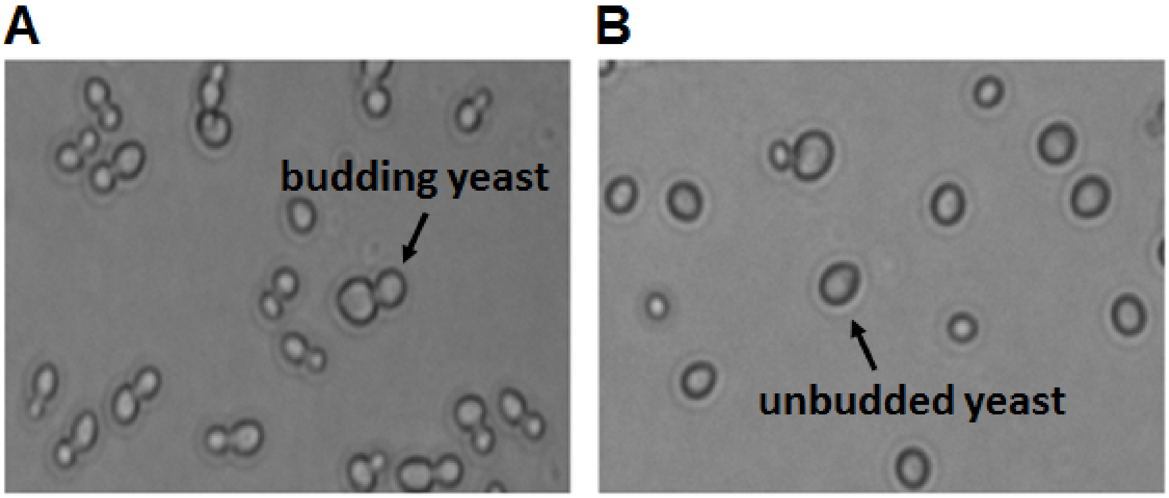

Figure S4. **The morphology of wide type yeast strain.** (A) The morphology of budding yeast cells in YPD media. (B) The morphology of unbuddedstationary-phase cellsin YP medium containing 2% raffinose.
