# Supplementary material for: Characteristics of replication-independent endogenous double-strand breaks in Saccharomyces cerevisiae
Source: BMC Genomics. 2014 Sep 1;15(1):750. doi: 10.1186/1471-2164-15-750 (PMC4158086; doi:10.1186/1471-2164-15-750)
Supplement: Supplementary file 7 — Additional file 7: Toy examples for counting the occurrences in the stitched read, genome, and contingency tables. (PDF 119 KB) [file 12864_2014_6418_MOESM7_ESM.pdf]

Count occurrences in stitched read

Suppose we have 5 stitched reads of length 100 bases. The below table show sequences only from position 45-55. Break position are between position 50 and 51.

			Base Position									
		45	46	47	48	49	50	break	51	52	53	54	55
Stitched Read ID	1	A	T	A	A	A	A	▼	C	G	G	T	C
	2	A	A	A	C	G	T	▼	G	G	T	T	A
	3	C	G	T	C	T	C	▼	C	C	A	A	G
	4	G	T	A	T	G	C	▼	G	T	A	C	C
	5	T	C	A	C	G	T	▼	A	A	A	T	A

The corresponding bases of each read for "xx▼xx", "xxx▼x", "xxxx▼", "xxxx▼x", "xxxx▼xx", "xxxxxx▼", "xxxxxx▼xx" are as below.

	Stitched Read ID				
	1	2	3	4	5
xx▼xx	AA▼CG	GT▼GG	TC▼CC	GC▼GT	GT▼AA
xxx▼x	AAA▼C	CGT▼G	CTC▼C	TGC▼G	CGT▼A
xxxx▼	AAAA▼	ACGT▼	TCTC▼	ATGC▼	ACGT▼
xxxx▼x	AAAA▼C	ACGT▼G	TCTC▼C	ATGC▼G	ACGT▼A
xxxx▼xx	AAAA▼CG	ACGT▼GG	TCTC▼CC	ATGC▼GT	ACGT▼AA
xxxxxx▼	ATAAAA▼	AAACGT▼	CGTCTC▼	GTATGC▼	TCACGT▼
xxxxxx▼xx	ATAAAA▼CG	AAACGT▼GG	CGTCTC▼CC	GTATGC▼GT	TCACGT▼AA

Number of occurrences of xxxx▼ in stitched reads are as below

4-mer	Counts	4-mer	Counts	4-mer	Counts	4-mer	Counts	4-mer	Counts	4-mer	Counts	4-mer	Counts	4-mer	Counts
AAAA	1	AGAA	0	CAAA	0	CGAA	0	GAAA	0	GGAA	0	TAAA	0	TGAA	0
AAAC	0	AGAC	0	CAAC	0	CGAC	0	GAAC	0	GGAC	0	TAAC	0	TGAC	0
AAAG	0	AGAG	0	CAAG	0	CGAG	0	GAAG	0	GGAG	0	TAAG	0	TGAG	0
AAAT	0	AGAT	0	CAAT	0	CGAT	0	GAAT	0	GGAT	0	TAAT	0	TGAT	0
AACA	0	AGCA	0	CACA	0	CGCA	0	GACA	0	GGCA	0	TACA	0	TGCA	0
AACC	0	AGCC	0	CACC	0	CGCC	0	GACC	0	GGCC	0	TACC	0	TGCC	0
AACG	0	AGCG	0	CACG	0	CGCG	0	GACG	0	GGCG	0	TACG	0	TGCG	0
AACT	0	AGCT	0	CACT	0	CGCT	0	GACT	0	GGCT	0	TACT	0	TGCT	0
AAGA	0	AGGA	0	CAGA	0	CGGA	0	GAGA	0	GGGA	0	TAGA	0	TGGA	0
AAGC	0	AGGC	0	CAGC	0	CGGC	0	GAGC	0	GGGC	0	TAGC	0	TGGC	0
AAGG	0	AGGG	0	CAGG	0	CGGG	0	GAGG	0	GGGG	0	TAGG	0	TGGG	0
AAGT	0	AGGT	0	CAGT	0	CGGT	0	GAGT	0	GGGT	0	TAGT	0	TGGT	0
AATA	0	AGTA	0	CATA	0	CGTA	0	GATA	0	GGTA	0	TATA	0	TGTA	0
AATC	0	AGTC	0	CATC	0	CGTC	0	GATC	0	GGTC	0	TATC	0	TGTC	0
AATG	0	AGTG	0	CATG	0	CGTG	0	GATG	0	GGTG	0	TATG	0	TGTG	0
AATT	0	AGTT	0	CATT	0	CGTT	0	GATT	0	GGTT	0	TATT	0	TGTT	0
ACAA	0	ATAA	0	CCAA	0	CTAA	0	GCAA	0	GTAA	0	TCAA	0	TTAA	0
ACAC	0	ATAC	0	CCAC	0	CTAC	0	GCAC	0	GTAC	0	TCAC	0	TTAC	0
ACAG	0	ATAG	0	CCAG	0	CTAG	0	GCAG	0	GTAG	0	TCAG	0	TTAG	0
ACAT	0	ATAT	0	CCAT	0	CTAT	0	GCAT	0	GTAT	0	TCAT	0	TTAT	0
ACCA	0	ATCA	0	CCCA	0	CTCA	0	GCCA	0	GTCA	0	TCCA	0	TTCA	0
ACCC	0	ATCC	0	CCCC	0	CTCC	0	GCCC	0	GTCC	0	TCCC	0	TTCC	0
ACCG	0	ATCG	0	CCCG	0	CTCG	0	GCCG	0	GTCG	0	TCCG	0	TTCG	0
ACCT	0	ATCT	0	CCCT	0	CTCT	0	GCCT	0	GTCT	0	TCCT	0	TTCT	0
ACGA	0	ATGA	0	CCGA	0	CTGA	0	GCGA	0	GTGA	0	TCGA	0	TTGA	0
ACGC	0	ATGC	1	CCGC	0	CTGC	0	GCGC	0	GTGC	0	TCGC	0	TTGC	0
ACGG	0	ATGG	0	CCGG	0	CTGG	0	GCGG	0	GTGG	0	TCGG	0	TTGG	0
ACGT	2	ATGT	0	CCGT	0	CTGT	0	GCGT	0	GTGT	0	TCGT	0	TTGT	0
ACTA	0	ATTA	0	CCTA	0	CTTA	0	GCTA	0	GTTA	0	TCTA	0	TTTA	0
ACTC	0	ATTC	0	CCTC	0	CTTC	0	GCTC	0	GTTC	0	TCTC	1	TTTC	0
ACTG	0	ATTG	0	CCTG	0	CTTG	0	GCTG	0	GTTG	0	TCTG	0	TTTG	0
ACTT	0	ATTT	0	CCTT	0	CTTT	0	GCTT	0	GTTT	0	TCTT	0	TTTT	0

Count occurrences in genome

Suppose reference genome has 2 chromosomes with the following sequences

chrI: ACGCTGATCGATCGTATCGAGC

chrII: TAGATCGATCGACTACGACTTATA

The xxxx occurrences in genome are count for each 4-mer. For example,

ACGC occur 1 times in the genome.

chrI: ACGCTGATCGATCGTATCGAGC

chrII: TAGATCGATCGACTACGACTTATA

ATCG occur 5 times in the genome.

chrI: ACGCTGATCGATCGTATCGAGC

chrII: TAGATCGATCGACTACGACTTATA

Number of occurrences of xxxx in the genome are as below

4-mer	Counts	4-mer	Counts	4-mer	Counts	4-mer	Counts	4-mer	Counts	4-mer	Counts	4-mer	Counts	4-mer	Counts
AAAA	0	AGAA	0	CAAA	0	CGAA	0	GAAA	0	GGAA	0	TAAA	0	TGAA	0
AAAC	0	AGAC	0	CAAC	0	CGAC	2	GAAC	0	GGAC	0	TAAC	0	TGAC	0
AAAG	0	AGAG	0	CAAG	0	CGAG	1	GAAG	0	GGAG	0	TAAG	0	TGAG	0
AAAT	0	AGAT	1	CAAT	0	CGAT	2	GAAT	0	GGAT	0	TAAT	0	TGAT	1
AACA	0	AGCA	0	CACA	0	CGCA	0	GACA	0	GGCA	0	TACA	0	TGCA	0
AACC	0	AGCC	0	CACC	0	CGCC	0	GACC	0	GGCC	0	TACC	0	TGCC	0
AACG	0	AGCG	0	CACG	0	CGCG	0	GACG	0	GGCG	0	TACG	1	TGCG	0
AACT	0	AGCT	0	CACT	0	CGCT	1	GACT	2	GGCT	0	TACT	0	TGCT	0
AAGA	0	AGGA	0	CAGA	0	CGGA	0	GAGA	0	GGGA	0	TAGA	1	TGGA	0
AAGC	0	AGGC	0	CAGC	0	CGGC	0	GAGC	1	GGGC	0	TAGC	0	TGGC	0
AAGG	0	AGGG	0	CAGG	0	CGGG	0	GAGG	0	GGGG	0	TAGG	0	TGGG	0
AAGT	0	AGGT	0	CAGT	0	CGGT	0	GAGT	0	GGGT	0	TAGT	0	TGGT	0
AATA	0	AGTA	0	CATA	0	CGTA	1	GATA	0	GGTA	0	TATA	1	TGTA	0
AATC	0	AGTC	0	CATC	0	CGTC	0	GATC	4	GGTC	0	TATC	1	TGTC	0
AATG	0	AGTG	0	CATG	0	CGTG	0	GATG	0	GGTG	0	TATG	0	TGTG	0
AATT	0	AGTT	0	CATT	0	CGTT	0	GATT	0	GGTT	0	TATT	0	TGTT	0
ACAA	0	ATAA	0	CCAA	0	CTAA	0	GCAA	0	GTAA	0	TCAA	0	TTAA	0
ACAC	0	ATAC	0	CCAC	0	CTAC	1	GCAC	0	GTAC	0	TCAC	0	TTAC	0
ACAG	0	ATAG	0	CCAG	0	CTAG	0	GCAG	0	GTAG	0	TCAG	0	TTAG	0
ACAT	0	ATAT	0	CCAT	0	CTAT	0	GCAT	0	GTAT	1	TCAT	0	TTAT	1
ACCA	0	ATCA	0	CCCA	0	CTCA	0	GCCA	0	GTCA	0	TCCA	0	TTCA	0
ACCC	0	ATCC	0	CCCC	0	CTCC	0	GCCC	0	GTCC	0	TCCC	0	TTCC	0
ACCG	0	ATCG	5	CCCG	0	CTCG	0	GCCG	0	GTCG	0	TCCG	0	TTCG	0
ACCT	0	ATCT	0	CCCT	0	CTCT	0	GCCT	0	GTCT	0	TCCT	0	TTCT	0
ACGA	1	ATGA	0	CCGA	0	CTGA	1	GCGA	0	GTGA	0	TCGA	4	TTGA	0
ACGC	1	ATGC	0	CCGC	0	CTGC	0	GCGC	0	GTGC	0	TCGC	0	TTGC	0
ACGG	0	ATGG	0	CCGG	0	CTGG	0	GCGG	0	GTGG	0	TCGG	0	TTGG	0
ACGT	0	ATGT	0	CCGT	0	CTGT	0	GCGT	0	GTGT	0	TCGT	1	TTGT	0
ACTA	1	ATTA	0	CCTA	0	CTTA	1	GCTA	0	GTTA	0	TCTA	0	TTTA	0
ACTC	0	ATTC	0	CCTC	0	CTTC	0	GCTC	0	GTTC	0	TCTC	0	TTTC	0
ACTG	0	ATTG	0	CCTG	0	CTTG	0	GCTG	1	GTTG	0	TCTG	0	TTTG	0
ACTT	1	ATTT	0	CCTT	0	CTTT	0	GCTT	0	GTTT	0	TCTT	0	TTTT	0

Contingency table

For each combination of xxxx, we construct a contingency table. For example, a contingency table for ACGT is as below.

	ACGT	the rest
reads	A= number of stitched reads that contain ACGT	B= number of stitched reads that had other sequences besides ACGT
genome	C= number of positions in the genome that are ACGT	D= number of positions in the genome that are other 4-mer sequence besides ACGT

Based on the toy data in the preceding section, a contingency table for ACGT is as below.

	ACGT	the rest
reads	A=2	B=3
genome	C=0	D=40
